# Demographic and socio-economic predictors of physical activity among people living with HIV of low socio-economic status

**DOI:** 10.4102/hsag.v24i0.1127

**Published:** 2019-10-24

**Authors:** Smart Z. Mabweazara, L.L. Leach, Clemens Ley, Sunday O. Onagbiye, Joel A. Dave, Naomi S. Levitt, Estelle V. Lambert

**Affiliations:** 1 Department of Sports, Recreation and Exercise Science, Faculty of Community and Health Sciences, University of the Western Cape, Cape Town, South Africa; 2 Department of Health Science, University of Applied Sciences, Vienna, Austria; 3 Department of Medicine, Faculty of Health Sciences, Division of Diabetic Medicine and Endocrinology, University of Cape Town, Cape Town, South Africa; 4 Department of Human Biology, Faculty of Health Sciences, University of Cape Town, Cape Town, South Africa

**Keywords:** physical activity, exercise, socio-economic status, HIV, AIDS

## Abstract

**Background:**

Physical activity (PA) is beneficial for the health of people living with HIV and AIDS (PLWHA).

**Aim:**

The aim of this study was to determine if age, body weight, height, gender, waist-to-hip ratio (WHR), educational attainment, employment status, CD4+ cell count and body mass index (BMI) can predict overall PA among PLWHA of low socio-economic status (SES).

**Setting:**

Participants in this study were HIV-infected patients on first-line antiretroviral therapy (ART) regimen offered by the South African National Department of Health, and those not on ART. Participants were conveniently sampled from a list at a community health care centre in Cape Town.

**Methods:**

This study sample consisted of 978 HIV-infected South Africans. Physical activity data were collected using the Global Physical Activity Questionnaire. Backward multiple linear regression modelling was used to determine the relative influence of variables (age, body weight, height, gender, WHR, educational attainment, employment status, CD4+ count and BMI) on total moderate-to-vigorous PA. Alpha level was set at 0.05.

**Results:**

The mean age of the participants was 38.2 (standard deviation [SD] = 8.76) years for men and 33.9 (SD = 8.53) years for women. Physical activity was significantly higher in men (480.2 [SD = 582.9] min/week) than among women (369.35 [SD = 222.53] min/week). The results of the multiple linear regression showed that educational attainment (β = 0.127; *p* = 0.00), employment (β = −0.087; *p* = 0.01) and gender (β = 0.235; *p* = 0.00) significantly predicted total moderate-to-vigorous PA. Gender had the greatest effect, followed by educational attainment and employment status.

**Conclusion:**

There is a need for PA programmes that are designed to (1) target women, (2) strengthen programmes for education and promotion of PA and (3) engage the unemployed into PA for PLWHA. Physical activity interventions for this particular group should be tailored for persons of low SES.

## Introduction

Physical activity (PA) is defined as any bodily movement produced by skeletal muscles that cause the utilisation of energy (Caspersen, Powell & Christenson 1985). Physical activity plays an important protective and therapeutic role in the prevention and management of chronic illnesses, such as cardiovascular disease (Sygit, Sygit & Pietrzak 2016). Physical activity has also been reported to be beneficial for numerous physiological body systems and can lessen most of the risk factors for non-communicable diseases (Cooper & Hancock 2012).

It has been suggested that PA is a cost-effective approach to prevent and manage chronic diseases (Giannini, Mohn & Chiarelli 2006). This finding has particular implications for chronic conditions, such as HIV and AIDS, that burden third-world economies and where pharmacological therapies may be associated with exorbitant financial costs and potential toxicities related to treatment (Fillipas et al. 2013). Moreover, evidence has shown that PA is an effective complementary therapy for managing HIV and/or AIDS (Jaggers & Hand 2016).

Given that PA is a cost-effective adjunctive therapy for the management of HIV and/or AIDS, and that persons of low socio-economic status (SES) are disproportionately burdened by HIV/AIDS (Ogunmola, Oladosu & Olamoyegun 2014), it presents the potential to be used to bridge the socio-economic disparities of health for people living with HIV and AIDS (PLWHA) of low SES. Understanding the physical and socio-demographic predictors of PA among PLWHA can be a first step towards understanding the factors that affect their PA behaviour. The knowledge generated from such research can be used in the design of contextualised PA interventions for PLWHA of low SES. Given the foregoing, the primary aim of this study was to determine if age, body weight, height, gender, waist-to-hip ratio (WHR), educational attainment, employment status, CD4+ cell count and body mass index (BMI) can predict overall PA among PLWHA.

## Theory

Individuals of low SES, especially those living in informal settlements or townships, have the highest prevalence of HIV in South Africa (Shisana & Simbayi 2002). One of the principal causes of mortality among PLWHA is co-morbid chronic cardio-metabolic diseases (Antiretroviral Therapy Cohort Collaboration 2010). Low SES is known to exacerbate these co-morbid conditions, in part, because of poor diet and low levels of PA, resulting in increased visceral fat mass accumulation and resultant obesity (Jaggers & Hand 2014). Evidence suggests that PA is a relatively inexpensive and effective complementary therapy for PLWHA (Jaggers & Hand 2016). This may be especially important for persons of low SES, who are less likely to engage in PA (Brennan, Brownson & Hovmand 2012). These individuals generally have poorer health status and more chronic disease risk factors (Kawachi, Kennedy & Wilkinson 1999).

People living with HIV and AIDS experience the adverse effects of pharmacological therapy, which negatively affects fat metabolism (Grunfeld et al. 2010). As such, PLWHA are reported to have significantly more abdominal fat accumulation than age-matched HIV-uninfected individuals (Shah et al. 2012). Central obesity has become a major concern among PLWHA, especially among women (Amorosa et al. 2005). Yahiaoui and Voss (2015) reported that it is necessary to understand the role of PA in minimising or preventing obesity among PLWHA. This is of particular significance among PLWHA of low SES, who are at a greater risk of being obese, especially women (McLaren 2007). The harmful effects of antiretroviral therapy and the increased incidence of obesity among PLWHA call for the use of complementary therapies, such as actively engaging in PA, to manage the resultant metabolic abnormalities (Yahiaoui & Voss 2015). To design PA interventions that address this concern, there is a need to understand the relationship between age, body weight, height, WHR, BMI and PA among PLWHA of low SES.

Resistance exercise has been found to be both safe and effective for improving muscle strength and body composition among PLWHA (Yarasheski et al. 2001), and low-intensity aerobic exercise has been associated with significant improvements in cardio-respiratory fitness and serum high-density lipoprotein cholesterol in the same population (Jaggers & Hand 2016).

Importantly, there is a need to understand factors that affect the PA behaviours of PLWHA to be able to design informed and context-sensitive PA interventions. There are, however, few quality studies concerning PA in HIV-positive persons, especially of low SES. Therefore, it is particularly important to understand the socio-economic correlates of PA for PLWHA of low SES because HIV and/or AIDS is a disease that is embedded in social and economic inequities (Perry 1998). Therefore, the relationship between employment status and health is an important matter (Bureau of Labor Statistics, U.S. Department of Labor 2018).

In terms of socio-demographic variables, Webel et al. (2015) examined the influence of gender and age among PLWHA and reported that middle-aged men are more likely to engage in PA than women. In the general population, unemployed individuals have been reported to be less physically active than employed individuals (Macassa et al. 2016). Non-HIV-infected persons with higher education levels are reported to have more opportunities of engaging in PA (Brown & Roberts 2011). With regard to physical variables, Smith et al. (2001) reported that aerobic exercise training safely lowers weight, BMI and abdominal girth in PLWHA. Mustafa et al. (1999) reported that exercising HIV-positive homosexual men showed an increase in CD4+ cell count. However, Smith et al. (2001) found no significant differences in CD4+ cell count between an exercising and non-exercising group of PLWHA.

In spite of a growing emphasis on the importance of PA in promoting health, PA among PLWHA in South Africa remains poorly researched. To the best of our knowledge, no study has investigated the influence of demographic and socio-economic determinants of PA among PLWHA of low SES.

## Materials and methods

### Sampling and participants

The present study is a secondary analysis utilising data from a larger cross-sectional investigation (Dave et al. 2011). In the primary study, convenient sampling was used to select HIV-infected patients at a community health clinic in Cape Town, South Africa. To be included in the study, participants needed to be 18 years or older and to not have changed their antiretroviral therapy (ART) within the past 6 months. Inclusion and exclusion criteria are reported in detail in Dave et al. (2011). The Research Ethics Committee of the Faculty of Health Sciences at the University of the Western Cape approved the secondary study (Ref. number: 14/10/33). Before participating in the study, procedures and risks were explained to the participants. Participants then gave their written informed consent to participate in the study. A total of 1035 patients consented to participate in the primary study. The present study made use of 978 participants, and excluded the participants with missing PA data.

### Data collection

Trained fieldworkers administered a researcher-generated questionnaire to the participants to obtain data on socio-demographic details, known diabetes risk factors, family history, medical history, smoking, alcohol and current medication. Subjects’ clinical records were reviewed, and information was extracted on prior weight, ART regimen, time on ART, CD4+ count, viral load, renal function, oral glucose tolerance test (OGTT), blood pressure measures, lipodystrophy and neuropathy examination. The clinical status and anthropometry data have been reported previously (Dave et al. 2011).

### Measures

#### Physical activity

The PA data reported in this study were collected using the Global Physical Activity Questionnaire (GPAQ). The GPAQ consists of 16 questions designed to estimate an individual’s level of PA in three domains (work, transport and leisure time) and time spent in sedentary behaviour (Bull, Maslin & Armstrong 2009). The GPAQ is a valid and reliable instrument for assessing PA among adults (Bull et al. 2009).

### Data analysis

Data were analysed using the Statistical Package for Social Science (SPSS) version 23 (IBM, New York, USA). Frequency distributions were calculated for all predictors and the outcome variable. Sub-categories related to ‘employment’ were dummy coded to produce two groups that were coded as ‘1’ (employed: inclusive of employed individuals and full-time homemakers) and ‘0’ (unemployed: inclusive of unemployed individuals, pensioners and those on a disability grant). Educational attainment was coded as follows: (1) never went to school; (2) up to grade 7/primary schooling; (3) grades 8–10/form 1–4; (4) grades 11–12/form 5–6 and (5) tertiary/diploma.

Participants’ age, gender, educational attainment, employment status, body weight, height, WHR, CD4+ cell count and BMI were considered as potential predictors for PA. An independent sample *t*-test was first performed to determine the significant differences in the variables between men and women. Spearman’s correlation analysis was then used to determine relationships between body weight, BMI, WHR, age, CD4+ cell count, gender and total moderate-to-vigorous PA. Correlations were interpreted using Dancey and Reidy’s (2004) categorisation criteria. Backward multiple linear regression modelling was used to determine the relative influence of variables (age, body weight, height, gender, WHR, educational attainment, employment status, CD4+ cell count and BMI) on total moderate-to-vigorous PA. Alpha level was set at 0.05.

### Ethical consideration

Permission to conduct the study was granted by the Senate Research Ethics Committee of the University of the Western Cape (registration number: 14/10/33).

## Results and findings

### Sample characteristics

The study sample consisted of 978 PLWHA. The mean age of the participants was 38.2 (standard deviation [SD] = 8.76) years for men and 33.9 (SD = 8.53) years for women. In terms of educational attainment, the average for the group was grades 11–12/form 5–6 (3.12 [SD = 0.92]). On average, most of the participants were employed (1.74 [SD = 0.48]). Women were significantly heavier in terms of BMI (27.5 [SD = 6.25] kg/m²) than their male counterparts (22.6 [SD = 3.46] kg/m²). CD4^+^ cell count was higher in women (369.35 [SD = 222.53] cells/µL) than in men (312.31 [SD = 234.27] cells/µL). Total moderate-to-vigorous PA in men (480.2 [SD = 582.9] min/week) was significantly higher than that of women (369.35 [SD = 222.53] min/week). Table 1 shows the physical characteristics of the participants.

**TABLE 1:** Physical characteristics of the participants mean (SD) (*N* = 978).

Variables	Men			Women			Total			*p*
	*N*	Mean	SD	*N*	Mean	SD	*N*	Mean	SD	
Age (year)	218	38.2	8.76	760	33.9	8.53	978	34.9	8.76	0.000***
Height (m)	215	1.69	0.06	749	1.57	0.06	964	1.60	0.07	0.000***
Weight (kg)	216	65.2	11.0	748	68.7	16.5	964	67.9	15.2	0.000***
BMI (kg/m²)	215	22.6	3.46	748	27.5	6.25	963	26.4	6.10	0.000***
WHR	213	0.90	0.06	742	0.84	0.11	955	0.85	0.11	0.000***
CD4+	185	312.31	234.27	673	369.35	222.53	858	357.0	226.2	0.003**
TMVPA	218	480.2	582.9	760	269.0	331.5	978	316.1	410.6	0.000***

SD, standard deviation; TVMPA, total vigorous-to-moderate physical activity; BMI, body mass index; WHR, waist-to-hip ratio.

** , Significant *p* =0.05

*** , Significant *p* = 0.01

### Correlation analysis

The bivariate correlation analysis showed that there was a significant relationship between total moderate-to-vigorous PA and height (*r* = 0.173, *p* = 0.000, *n* = 964), and an inverse relationship for employment (*r* = −0.132, *p* = 0.000, *n* = 978). The results also showed that there was no significant correlation between total moderate-to-vigorous PA and age (*r* = 0.002, *p* = 0.955, *n* = 978), CD4+ count (*r* = −0.013, *p* = 712, *n* = 858), body weight (*r* = −0.004, *p* = 0.907, *n* = 964), BMI (*r* = −0.074, *p* = 0.021, *n* = 963), WHR (*r* = 0.019, *p* = 0.553, *n* = 955) and education (*r* = 0.095, *p* = 0.003, *n* = 978). Table 2 shows the Pearson correlation matrix between body weight, BMI, WHR, age, CD4^+^ count, gender and total moderate-to-vigorous PA.

**TABLE 2:** Pearson correlation matrix showing relationships between body weight, body mass index, waist-to-hip ratio, age, CD4 count, gender and moderate-to-vigorous physical activity (*N* = 978).

Variables	Age	CD4+	Weight	Height	BMI	WHR	Education	Employment	TMVPA
**Age**									
Pearson correlation	1	−0.038	0.092**	0.073*	0.067*	0.204**	−0.403**	0.025	0.002
Sig. (2-tailed)	-	0.272	0.004	0.024	0.036	0.000	0.000	0.439	0.955
*N*	978	858	964	964	963	955	978	978	978
**CD4+ count**									
Pearson correlation	−0.038	1	0.138**	−0.046	0.157**	0.104**	0.020	−0.027	−0.013
Sig. (2-tailed)	0.272	-	0.000	0.184	0.000	0.003	0.558	0.431	0.712
*N*	858	858	847	847	846	838	858	858	858
**Body weight**									
Pearson correlation	0.092**	0.138**	1	0.215**	0.909**	0.154**	0.071*	−0.106**	−0.004
Sig. (2-tailed)	0.004	0.000	-	0.000	0.000	0.000	0.027	0.001	0.907
*N*	964	847	964	963	963	955	964	964	964
**Height**									
Pearson correlation	0.073*	−0.046	0.215**	1	−0.201**	0.133**	−0.023	−0.119**	0.173**
Sig. (2-tailed)	0.024	0.184	0.000	-	0.000	0.000	0.471	0.000	0.000
*N*	964	847	963	964	963	954	964	964	964
**BMI**									
Pearson correlation	0.067*	0.157**	0.909**	−0.201**	1	0.100**	0.073*	−0.051	−0.074*
Sig. (2-tailed)	0.036	0.000	0.000	0.000	-	0.002	0.024	0.114	0.021
*N*	963	846	963	963	963	954	963	963	963
**WHR**									
Pearson correlation	0.204**	0.104**	0.154**	0.133**	0.100**	1	−0.139**	−0.005	0.019
Sig. (2-tailed)	0.000	0.003	0.000	0.000	0.002	-	0.000	0.876	0.553
*N*	955	838	955	954	954	955	955	955	955
**Education**									
Pearson correlation	−0.403**	0.020	0.071*	−0.023	0.073*	−0.139**	1	−0.114**	0.095**
Sig. (2-tailed)	0.000	0.558	0.027	0.471	0.024	0.000	-	0.000	0.003
*N*	978	858	964	964	963	955	978	978	978
**Employment**									
Pearson correlation	0.025	−0.027	−0.106**	−0.119**	−0.051	−0.005	−0.114**	1	−0.132**
Sig. (2-tailed)	0.439	0.431	0.001	0.000	0.114	0.876	0.000	-	0.000
*N*	978	858	964	964	963	955	978	978	978
**TMVPA**									
Pearson correlation	0.002	−0.013	−0.004	0.173**	−0.074*	0.019	0.095**	−0.132**	1
Sig. (2-tailed)	0.955	0.712	0.907	0.000	0.021	0.553	0.003	0.000	-
*N*	978	858	964	964	963	955	978	978	978

TMVPA, total moderate-to-vigorous physical activity; BMI, body mass index; WHR, waist-to-hip ratio.

* , Correlation is significant at the 0.05 level (2-tailed).

** , Correlation is significant at the 0.01 level (2-tailed).

### Regression analysis

The results of the multiple linear regression analysis to predict total moderate-to-vigorous PA using age, CD4+ count, gender, height, body weight, BMI, WHR, education and employment showed that education, employment and gender significantly predicted total moderate-to-vigorous PA (Table 3). Education, employment status and gender accounted for 7.8% (*R*^2^ = 0.078; *p* = 0.000) of the variance. The generalised equation used to predict total moderate-to-vigorous PA from education, employment and gender was: total moderate-to-vigorous PA = 216.143 + 56.599 (education) – 73.465 (employment) – 235.485 (gender: 1 = men, 0 = women) obtained from the coefficients table. Education, employment and gender significantly predicted total vigorous-to-moderate physical activity (*F* [9.827] = 7.734, *p* < 0.001, *R*^*2*^ = 0.068 at *p* < 0.05). The results of the regression analysis showed that being male was predictive of increased total moderate-to-vigorous PA. Males had 235.485 more minutes of total moderate-to-vigorous PA per week than females. Being employed was also predictive of increased total moderate-to-vigorous PA. Unemployed individuals had 73.469 less minutes of total moderate-to-vigorous PA than employed individuals. Educational attainment level was also predictive of total moderate-to-vigorous PA. An increase in educational attainment meant an increase in total moderate-to-vigorous PA by 56.599 minutes per week. Gender had the greatest effect (β = 0.235; *p* = 0.00), followed by educational attainment (β = 0.127; *p* = 0.00) and employment (β = −0.087; *p* = 0.01). Body mass index, height, WHR, CD4+ count and age did not predict total moderate-to-vigorous PA.

**TABLE 3:** Multiple linear regression analysis to predict total moderate vigorous physical activity and body weight, body mass index, waist-to-hip ratio, age, CD4 count and gender (*N* = 978).

Variable	Regression coefficients					Model summary					
	*B*	SE	β	*t*	*p*	*R*	*R*^2^	*F*	*df*_1_	*df*_2_	*p*
Criterion: TMVPA	-	-	-	-	-	0.274	0.075	22.529	3	833	0.000
Intercept	216.143	76.752		2.816	0.005	-	-	-	-	-	-
Education	56.599	15.298	0.127	3.700	0.000	-	-	-	-	-	-
Employment	−73.469	28.486	−0.087	−2.579	0.010	-	-	-	-	-	-
Gender	235.485	34.439	0.235	6.838	0.000	-	-	-	-	-	-

TMVPA, total moderate-to-vigorous physical activity.

*p* < 0.05.

## Discussion

The aim of this study was to establish if age, body weight, height, gender, WHR, educational attainment, employment status, CD4+ cell count and BMI predicted PA among PLWHA of low SES. It was found that education, employment status and gender significantly predicted total moderate-to-vigorous PA.

Gender was found to have a greater effect on total moderate-to-vigorous PA than education and employment. Webel et al. (2015) examined patterns of free-living exercise in adults with HIV by gender and age and reported that middle-aged men exercised more regularly than women. Similarly, an earlier study indicated that men are more likely to engage in PA than women (Lichtman et al. 1992). Married couples and females, in general, engage in less exercise than males (Humphreys & Ruseski 2009). In African settings, men generally tend to be employed, providing for the whole family, while women are responsible for household chores and taking care of the children.

Currently, in South Africa, there is substantial infrastructural development and men of low SES are employed in work that characteristically requires high levels of moderate-to-vigorous PA (Malambo et al. 2016). Likewise, sociocultural factors in African communities impact in such a way that women are more likely to be employed in domestic work with less opportunities for engaging in health-promoting moderate-to-vigorous PA (Walter & Du Randt 2011). Furthermore, within the African culture, women tend to engage in hobbies that are often housebound, while men usually engage in physically demanding recreational activities (Walter & Randt 2011). The impact of these sociocultural factors in South Africa means that inadequate PA, particularly among women of low SES, poses a serious health risk (Malambo et al. 2016). Thus, there is a need for health-promoting PA interventions, specifically for women of low SES in all age groups (Strydom 2013). Bellows-Rieken and Rhodes (2008) noted that PA changes during parenthood may be moderated by gender, with mothers experiencing the largest decline, as women are traditionally the primary caregivers. Moreover, Verhoef and Love (1994) reported that the amount of leisure time available for women who are mothers was one of the most important predictors of participation in PA. The implication of this finding for PLWHA in low-income settings is that PA interventions should focus more on motivating women into participating in PA, and that women could gain more from PA interventions than men.

For employment, one might argue that the results may be confounded by clinical status. However, the present results showed no correlation between CD4+ cell count and employment. Employment was also found to be a predictor of PA. Similar results have been reported in other studies with non-HIV-infected persons (Macassa et al. 2016). Macassa et al. (2016) inquired into the dissimilarities in PA by employment status among economically active individuals. Unemployed individuals were more likely to be physically inactive when compared with their employed counterparts. Arguably, individuals who earn are generally better able to find time for PA (North Carolina Institute of Medicine Task Force on Prevention 2009), and they can afford to pay for being a member of a fitness or wellness club.

Health insurance and health care also become more accessible with the availability of monetary resources (North Carolina Institute of Medicine Task Force on Prevention 2009). On the contrary, individuals who are unemployed, especially those of a low-income background, are more likely to live in substandard housing or in unsafe communities, which restrict engagement in PA. These communities often lack access to outdoor recreational amenities where the residents can engage in PA.

Employed PLWHA of low SES are likely to afford health-enhancing opportunities (e.g. PA, a healthy diet and medical insurance), which often positively influence their quality of life, with regard to physical and mental health. In addition, job security might also enhance their self-esteem and self-efficacy to exercise, thus placing them in a better position to engage in PA. For employed PLWHA, job security is generally related to better mental quality of life (Rueda et al. 2012). Being employed may mean that they have to walk to-and-from work daily compared with those who are unemployed and more likely to be sedentary.

It has been reported that differences in income generally make the greatest disparities for health (North Carolina Institute of Medicine Task Force on Prevention 2009). Unemployment is viewed as a permanent stressor requiring individuals to adapt to the situation (Macassa et al. 2016). The unemployed are in a very difficult situation financially, which is exacerbated by their HIV status and resultant social marginalisation, which, in turn, lead to feelings of worthlessness (Kapuvári 2011). It is argued that the stress experienced by unemployed individuals might be the primary cause of their unhealthy behaviour (Macassa et al. 2016).

Educational attainment was a predictor of PA, with an increase in educational attainment associated with an increase in total moderate-to-vigorous PA. Brown and Roberts (2011) stated that higher levels of education increase the prospects of engaging in frequent PA. Shaw and Spokane (2008) reported that among adults with low levels of education, being unemployed was related to low levels of PA, whereas for those who were highly educated the reverse was true.

On the contrary, Frantz and Murenzi (2013) established that among PLWHA of distinctively lower education levels (i.e. 65% had only completed up to primary school), the majority were inactive and mainly engaged in leisure-time PA. Moreover, Hui, Hui and Xie (2014) reported that education level was positively related with PA knowledge among adults with type 2 diabetes. Educated individuals are in a position to understand the beneficial effects of PA for managing HIV and/or AIDS, and thus engage more in PA. Thus, a PA education programme may be more effective in promoting regular PA. This finding also reinforces the need to strengthen programmes that focus on education and the promotion of PA for PLWHA of low SES.

The findings of particular importance for this specific study were that for total moderate-to-vigorous PA, the predictors of PA, in order of their effect, were gender, educational attainment and employment. This finding reinforces the need to strengthen programmes of education and the promotion of PA. In addition, PA interventions should be aimed at creating conducive environments in the workplace that motivate people to engage in regular PA.

### Strengths

This study is one of the few that have examined the relationships between SES and PA among PLWHA of low SES in South Africa. The study explains how socio-demographic variables predict PA among PLWHA. This information may be used to design PA interventions that are aimed at engaging PLWHA of low SES in PA.

### Limitations

Use of different instruments in data collection hinders the comparison between studies. Furthermore, only a few studies have investigated the combined effects of demographic and socio-economic variables on PA among PLWHA of low SES, which further limits comparisons with other studies.

### Recommendations

Future researchers should focus their research on the demographic and socio-economic predictors of domain-specific PA among PLWHA of low SES. It is important for interventionists to understand how the socio-demographic determinants of PA influence specific PA domains in order for them to use specific techniques that target PA domains of interest. Studies should also attempt to understand how these predictors relate with different PA intensities (i.e. low, moderate or vigorous PA).

## Conclusions

The results of this study revealed that among PLWHA of low SES, gender, education and employment were significant predictors of PA. Accordingly, for this particular population, there is a need for PA programmes and policies that are designed to (1) target women, (2) strengthen programmes of education and the promotion of PA and (3) engage the unemployed in regular PA. Physical activity interventions for PLWHA should take cognisance of the health disparities that exist between persons of low SES and those of higher SES to be context sensitive.
