# Erratum: Demographic and socio-economic predictors of physical activity among people living with HIV of low socio-economic status

**DOI:** 10.4102/hsag.v26i0.1560

**Published:** 2021-07-29

**Authors:** Smart Z. Mabweazara, L.L. Leach, Clemens Ley, Sunday O. Onagbiye, Joel A. Dave, Naomi S. Levitt, Estelle V. Lambert

**Affiliations:** 1 Department of Sports, Recreation and Exercise Science, Faculty of Community and Health Sciences, University of the Western Cape, Cape Town, South Africa; 2 Department of Health Science, University of Applied Sciences FH Campus Wien Vienna, Austria; 3 Department of Medicine, Faculty of Health Sciences, Division of Diabetic Medicine and Endocrinology, University of Cape Town, Cape Town, South Africa; 4 Department of Human Biology, Faculty of Health Sciences, University of Cape Town, Cape Town, South Africa

In the version of this article initially published, Mabweazara, S.Z., Leach, L.L., Ley, C., Onagbiye, S.O., Dave, J.A., Levitt, N.S. et al., 2019, ‘Demographic and socio-economic predictors of physical activity among people living with HIV of low socio-economic status’, *Health SA Gesondheid* 24(0), a1127. https://doi.org/10.4102/hsag.v24i0.1127, the third author’s affiliation was given incorrectly. The correct affiliation should be ‘Department of Health Science, University of Applied Sciences FH Campus Wien, Vienna, Austria’ instead of ‘Department of Health Science, University of Applied Sciences, Vienna, Austria’.

This correction does not alter the study’s findings of significance or overall interpretation of the study’s results. The publisher apologises for any inconvenience caused.

